# VCF2CAPS–A high-throughput CAPS marker design from VCF files and its test-use on a genotyping-by-sequencing (GBS) dataset

**DOI:** 10.1371/journal.pcbi.1008980

**Published:** 2021-05-20

**Authors:** Wojciech Wesołowski, Beata Domnicz, Joanna Augustynowicz, Marek Szklarczyk

**Affiliations:** 1 Department of Plant Biology and Biotechnology, Faculty of Biotechnology and Horticulture, University of Agriculture in Krakow, Krakow, Poland; 2 Department of Botany, Physiology and Plant Protection, Faculty of Biotechnology and Horticulture, University of Agriculture in Krakow, Krakow, Poland; Johns Hopkins University, UNITED STATES

## Abstract

Next-generation sequencing (NGS) is a powerful tool for massive detection of DNA sequence variants such as single nucleotide polymorphisms (SNPs), multi-nucleotide polymorphisms (MNPs) and insertions/deletions (indels). For routine screening of numerous samples, these variants are often converted into cleaved amplified polymorphic sequence (CAPS) markers which are based on the presence versus absence of restriction sites within PCR products. Current computational tools for SNP to CAPS conversion are limited and usually infeasible to use for large datasets as those generated with NGS. Moreover, there is no available tool for massive conversion of MNPs and indels into CAPS markers. Here, we present VCF2CAPS–a new software for identification of restriction endonucleases that recognize SNP/MNP/indel-containing sequences from NGS experiments. Additionally, the program contains filtration utilities not available in other SNP to CAPS converters–selection of markers with a single polymorphic cut site within a user-specified sequence length, and selection of markers that differentiate up to three user-defined groups of individuals from the analyzed population. Performance of VCF2CAPS was tested on a thoroughly analyzed dataset from a genotyping-by-sequencing (GBS) experiment. A selection of CAPS markers picked by the program was subjected to experimental verification. CAPS markers, also referred to as PCR-RFLPs, belong to basic tools exploited in plant, animal and human genetics. Our new software–VCF2CAPS–fills the gap in the current inventory of genetic software by high-throughput CAPS marker design from next-generation sequencing (NGS) data. The program should be of interest to geneticists involved in molecular diagnostics. In this paper we show a successful exemplary application of VCF2CAPS and we believe that its usefulness is guaranteed by the growing availability of NGS services.

This is a *PLOS Computational Biology* Software paper.

## Introduction

Next-generation sequencing (NGS) technologies are powerful tools for in-depth genetic studies of even poorly characterized species. In such context, NGS is commonly used for *de novo* sequencing [1], quantitative analysis of gene expression by transcriptome sequencing [2], and for marker discovery [3]. NGS-based genotyping allows for massive detection of DNA sequence variants such as single nucleotide polymorphisms (SNPs), multi-nucleotide polymorphisms (MNPs) and insertions/deletions (indels). The unsurpassed efficiency of high-throughput DNA sequencing outcompeted earlier methods of marker discovery such as RAPDs, RFLPs, AFLPs, and even microarray-based platforms like e.g. Diversity Arrays Technology (DArT) [4,5]. At present NGS-based detection of sequence polymorphisms is possible through the use of whole genome re-sequencing as well as various methods of target capture and complexity reduction, with the latter being more and more popular due to its open character (adjustable number of analyzed sequence tags) and cost-effectiveness [6]. Complexity reduction (also referred to as reduced representation) is usually achieved through application of a restriction digest during preparation of sequencing libraries, as in the case of RAD-seq (restriction site-associated DNA sequencing, [7]) or GBS (genotyping-by-sequencing, [8]). In these contexts, sequencing is typically performed using various Illumina systems of which the most powerful generate data outputs at a terabase level (https://illumina.com). Despite the robustness of the NGS-driven discovery of sequence polymorphisms it is often desirable to convert them into conventional PCR-based markers being very useful in routine screening of numerous samples, even in standardly equipped laboratories. PCR detection of DNA polymorphisms offers several alternatives, among them cleaved amplified polymorphic sequence (CAPS) markers which are based on the presence versus absence of restriction sites within amplification products. Since their invention [9] CAPS markers have attracted substantial interest in genetic research, mostly due to their co-dominant character, methodological simplicity as well as ease of result interpretation [10]. It is worth mentioning that the term CAPS is used mostly by plant scientists while in human and animal genetics these markers are rather referred to as PCR-RFLPs–under this name they were recognized as the “gold standard” of molecular diagnostics [11].

There are several software tools that facilitate conversion of SNPs into CAPS markers: CAPS Designer [12], dCAPS [13], SNP2CAPS [14], BlastDigester [15], CapsID [16], and PCR Markers Tools [17]. However, most of them were designed to analyze relatively small numbers of sequences and cannot be readily scaled up to large volumes of NGS data. An exception is PCR Markers Tools, which is a set of scripts aimed at parsing variant output files, detecting restriction polymorphisms and designing PCR primers. These scripts were adapted for use within the Galaxy framework [18], making them available for scientists with lower bioinformatic expertise.

Our research purpose was to develop a convenient software for identification of CAPS markers from large-scale genotyping experiments as well as to test its functionality using an exemplary NGS dataset. In order to further expand and improve the high-throughput design of CAPS markers, we present here an open-source, freely available software–VCF2CAPS–which via a simple and intuitive Graphic User Interface (GUI) serves for identification of restriction endonucleases that recognize SNP/MNP/indel-containing sequences from NGS experiments. In addition to identification of CAPS markers, our software is provided with two useful filtration utilities not available in other SNP to CAPS converters: 1) filtering markers with a single polymorphic restriction site (so with only one cut or with no cut within a user-specified sequence length); this feature guarantees obtaining simple and easily interpretable restriction profiles (only 1–3-banded); 2) filtering markers that differentiate up to three user-defined groups of individuals or samples from the analyzed population; if such groups represent phenotypic classes, the use of this utility allows identification of CAPS markers which are linked to genes responsible for the observed phenotypic variation. Performance of VCF2CAPS was tested on a thoroughly analyzed dataset from a GBS experiment. The analyzed plant population (table beet) segregated with respect to a monogenic trait (fertility restoration). Using this population a set of theoretical (VCF2CAPS-picked) CAPS markers was subjected to laboratory verification showing general agreement between the *in silico* and electrophoretic data.

## Materials and methods

### VCF2CAPS development

VCF2CAPS was written using the Perl5 programming language (https://www.perl.com). The graphic user interface was developed using the Perl/Tk module, which together with other Perl modules (Digest::MD5, LWP::Simple, PAR::Packer) was installed from the Comprehensive Perl Archive Network (CPAN) (https://www.cpan.org)–a repository of software modules written in Perl. The CPAN client, used for downloading and installing Perl modules from the CPAN repository, was part of the Strawberry Perl (v.5.26.0.1-32bit) (http://strawberryperl.com)–a complete Perl environment for MS Windows. The executable version of our program for Windows operating systems was generated using the PAR::Packer module.

The MS Windows executable version has been tested with a number of computers and should work on most machines running MS Windows XP or later with a 32- or 64-bit CPU.

### Plant material

The seed of table beet mapping population– 398 –was produced in Plantico Zielonki Ltd. by self-pollination of an F_1_ plant obtained by crossing male-sterile line 4357A with the MO pollinator line. The seed was forwarded to KHNO Polan Ltd. where the population was cultivated until flowering. The fertility phenotype (male-sterile vs. male-fertile) of individual plants was determined using visual inspection and subsequently it was verified by microscopic examination of pollen subjected to Alexander’s stain [19].

### DNA sample preparation

Total genomic DNA was isolated from frozen (-80°C) leaves according to the procedure of Szklarczyk [20] scaled up to 1 g of the ground plant tissue. The final DNA pellet was dissolved in 200 μl of TE buffer and a 50 μl aliquot of the resulting sample was supplemented with 5 μl of RNase A (10 μg/ml) which was followed by 1 h incubation at 37°C. After digestion with RNase A, DNA samples were mixed with Membrane Binding Solution (1:1, v/v) from the Wizard SV Gel and PCR Clean-up System (Promega), and transferred to SV Minicolumns from the same kit. All the remaining steps of DNA purification were conducted according to the Promega protocol (Technical bulletin TB308).

### GBS library preparation and sequencing

Sequence data were generated by the Beijing Genomics Institute (BGI), Hong Kong, China. For library production the purified DNAs were digested with *Ape*KI. The library was subjected to Illumina PE100 sequencing. The raw sequence reads were submitted to the Sequence Read Archive (SRA, NCBI) under accession number PRJNA655397.

### Alignment of sequence reads to the reference genome and variant calling

The Illumina sequence reads were aligned to the sugar beet reference genome (GenBank: AYZS00000000.2) using the Burrows-Wheeler Aligner (BWA, [21]). The BWA output files, saved in the SAM (Sequence Alignment/Map) format, were converted to their binary versions–BAM files–using SAMtools [22]. Next, with the same software package the BAM files were sorted and indexed. Variant calling was performed using Platypus [23] with the following parameters: nCPU = 6, minMapQual = 20, minBaseQual = 20, minGoodQualBases = 5, badReadsThreshold = 10, rmsmqThreshold = 20, abThreshold = 0.01, maxReadLength = 250, hapScoreThreshold = 20, trimAdapter = 0, maxGOF = 20, minReads = 2, genIndels = 1, minFlank = 5, sbThreshold = 0.01, scThreshold = 0.95, hapScoreThreshold = 15, filterDuplicates = 0, filterVarsByCoverage = 0, filteredReadsFrac = 0.7, minVarFreq = 0.002, mergeClusteredVariants = 0, filterReadsWithUnmappedMates = 0.

The next step was filtering all bi-allelic variants that passed all filters during variant calling. This filtration was performed using VCFtools [24] with parameters: -- remove-filtered-all, -- min-alleles 2, -- max-alleles 2.

### Annotation of variants

Variant annotation was performed using ANNOVAR [25]. The annotation file of the sugar beet reference genome in the gff3 format was downloaded from https://www.ncbi.nlm.nih.gov/genome/?term=sugar+beet. The gff3 file was converted to the GenePred format using the gff3ToGenePred tool, available at http://hgdownload.soe.ucsc.edu/admin/exe/linux.x86_64/. Next, the obtained GenePred file was converted to the refGene format by adding the line number at the beginning of each line. The obtained annotation file for the reference genome (in the refGene format) was one of two files which created a database required by ANNOVAR. The second file contained FASTA-formatted sequences of coding regions included in the refGene file. This file was created with the retrieve_seq_from_fasta.pl tool (available in ANNOVAR) using the refGene file (see above) and the sugar beet reference genome as inputs. Finally, the variants obtained after initial filtration were annotated with the table_annovar.pl tool. The resulting output file was parsed with a custom Python script to extract annotation statistics.

### Extraction of CAPS markers

Before loading into VCF2CAPS, variants in the VCF file were subjected to the chi-square test (MS Excel 2007) to verify their segregation ratio. Variants (DNA polymorphisms) showing significant deviation from the 1:2:1 (a:h:b) ratio (*P*<0.05) or with more than 5 missing genotypes were excluded from the analysis. The search for CAPS markers was performed taking into account 619 commercially available restriction enzymes. The identified CAPS markers were subjected to filtration by groups (VCF2CAPS utility) to pick up markers that differentiated male-sterile and male-fertile plants. This filtration was performed allowing at most 10% of mismatches for both male-sterile and male-fertile plants.

### Genetic mapping

For map construction the variants (DNA polymorphisms) were used for which VCF2CAPS identified a polymorphic restriction site. At first, all CAPS markers were parsed with a custom AWK script to retain only those that were at least 10 kb apart one from another. This filtration was not applied to the markers from chromosome 7 which was the poorest with respect to the number of identified polymorphisms. Next, the markers were filtered using the R language [26] to remove those with duplicated segregation patterns. Linkage analysis and linear ordering of loci were performed using JoinMap 4.0 [27] with the regression mapping algorithm. Homozygotes were encoded as ‘a’ and ‘b’ for markers of maternal and paternal origin, respectively, while heterozygotes were encoded as ‘h’. Assuming that fertility restoration was conditioned by a single dominant gene, the genotype of male-sterile plants was encoded as ‘a’, while for male-fertile plants–as ‘c’ (not ‘a’). Linkage groups were created using a maximum recombination fraction of 0.4 and a minimum LOD score of 5. Maps distances in cM were calculated using Haldane’s mapping function. Chromosome numbers were assigned to the obtained linkage groups based on the genome location of the respective markers. The linkage groups were visualized with MapChart 2.2 [28].

### CAPS analysis

PCR primers were designed manually on the basis of the SNP/MNP/indel-containing sequences from the VCF2CAPS output file (Table 1). PCR was performed in a total volume of 15 μl containing 75 ng of genomic DNA, 1× PCR buffer (Dongsheng Biotech, Guangzhou, China), 5 mM MgCl_2_, 0.25 mM dNTPs, 0.25 μM each primer and 1.1 U of *Taq* DNA polymerase (Dongsheng Biotech). Temperature cycling was performed as follows: initial denaturation at 94°C for 5 minutes followed by 35 cycles of 92°C for 45 s, 57°C for 45 s and 72°C for 2 minutes, then a final extension at 72°C for 10 minutes.

**Table 1:** CAPS markers selected for experimental verification.

Marker designation		Chromosome/scaffold	Forward primer (5’->3’)	Reverse primer (5’->3’)	Enzyme
Original	Abbreviated				
**1–3936017**	1–017	1	TTCACACGCATCGCGTGCAT	AGAGATCGCTCACATGTACA	*Taq*I
**1–7702028**	1–028	1	CAGTTCATCAATCATCATAC	AATTGTTGCTATCAGATGCT	*Taq*I
**2–37666525**	2–525	2	CGTACTTACATATATGCTTG	ATGATCTTGATAGCCTAGCT	*Taq*I
**3–2542300**	3–300	3	TGTGTTCAGCTCCTCATTGC	GTGTGAACTCTGTCACATGT	*Ava*II
**3–2596351**	3–351	3	CTGACATAGAGTGAGTGTTC	TGTAACTATTGCAGTGAGCA	*Alw*I
**3–5163363**	3–363	3	TAGCAGGTCTGTCACTGATG	GCTTAAGATCTATGGTCAGT	*Taq*I
**3–2098579**	3–579	3	ATCCTTGAGTGCTCCAAGCA	GCTGTCACTTACTTCAGATC	*Ssp*I
**3–2549714**	3–714	3	TCACTGCAACAGACACAAGA	TTCAGTTACGCAGCGTGATC	*Bsi*WI
**6–6945022**	6–022	6	CGTGATGTAGTCTCAGATAG	TGCAGAAGTGATGTATCATG	*Taq*I
**0093.scaffold00331-195256**	s–256	0093.scaffold00331	CAGTCTTGTCCAGTTGCACA	ATACTAGTCTCACTATCAGT	*Taq*I

Twelve microliters of the post-PCR mixture were supplemented with 5 U of a restriction enzyme and incubated at the appropriate temperature for 16 hours. For enzymes *Alw*I, *Ava*II, *Bsi*WI, and *Ssp*I (New England Biolabs) restriction was carried out at 37°C, while for *Taq*I (Thermo Fisher Scientific) at 65°C. The resulting restriction fragments were separated in 1% agarose alongside with 5 μl of the 100 bp and 1 kb DNA ladders from Dongsheng Biotech. Prior to gel loading, 2 μl of the 6 × Loading Buffer (Dongsheng Biotech) were added to the post-restriction mixture. DNA electrophoresis was performed in the TBE buffer at a field strength of 4 V/cm for 2 hours. The gels contained ethidium bromide at a concentration of 1.33 μg/ml.

## Design and implementation

### Input data

**Fig 1:**
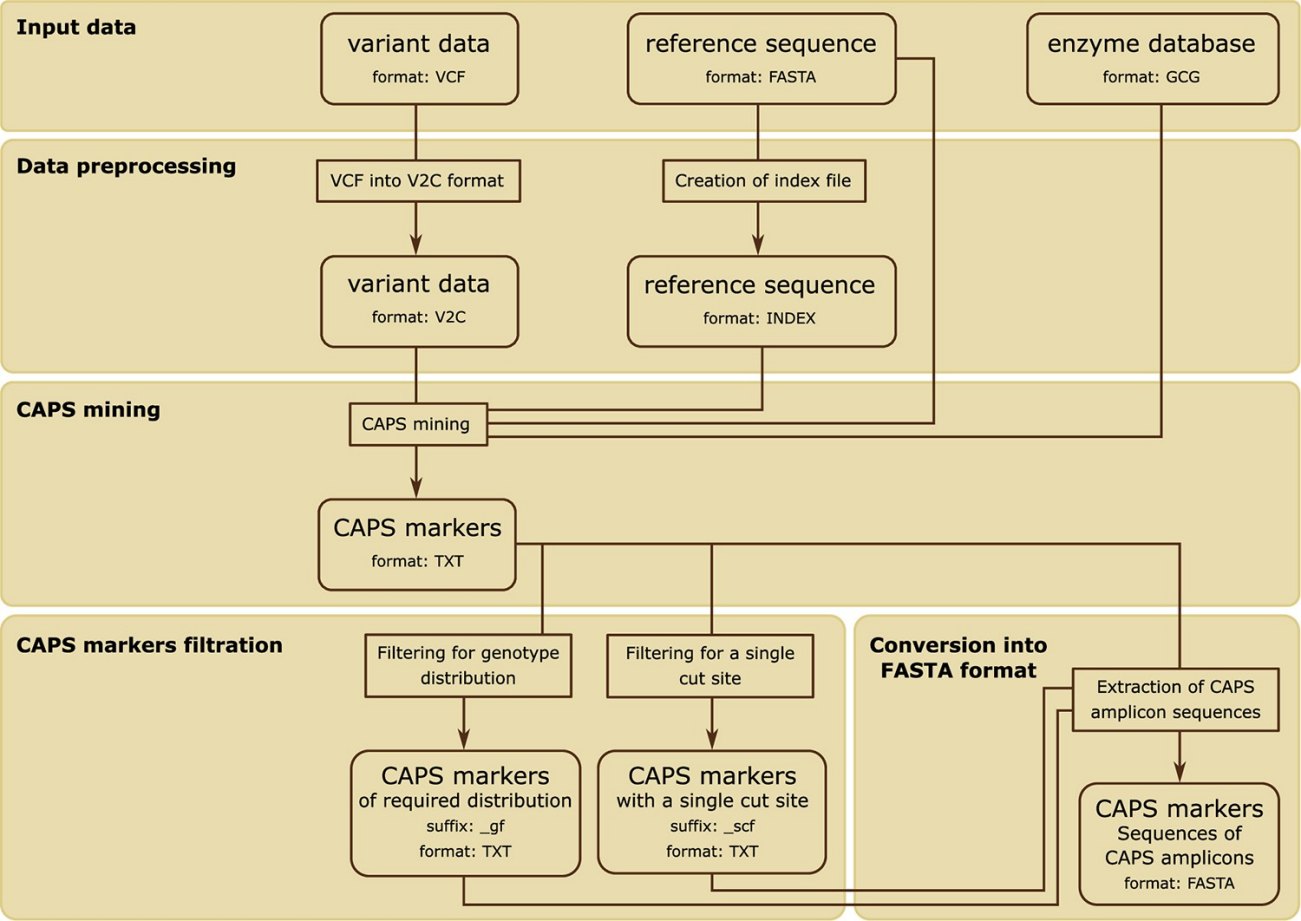
Visualization of the VCF2CAPS workflow.

The workflow of the VCF2CAPS program is shown in Fig 1. VCF2CAPS requires three input files. The first is a FASTA-formatted file containing the reference sequence that was used for short read alignment. The second file contains data of restriction enzymes in GCG format. It can be downloaded from the database of restriction enzymes–REBASE ([29], http://rebase.neb.com/rebase/link_gcg). The third input file is a VCF (Variant Call Format) file created by variant calling software.

### Data preprocessing

At the beginning of the analysis, VCF2CAPS creates an index file of the reference sequence, which is required for quick access to any sequence within the FASTA file. The index is a tab-delimited text file with the following five columns: 1) sequence name; 2) offset (in bytes) within the FASTA file of the sequence’s first base; 3) number of bases in each line except the last; 4) number of bases in the sequence’s last line; 5) sequence length (in bases).

**Fig 2:**
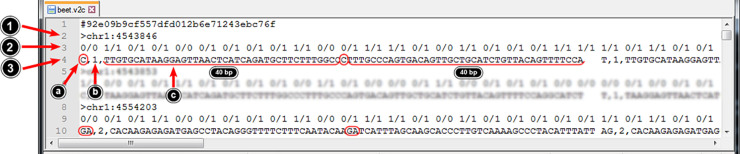
The V2C file format–file elements are marked with arrows and described in the text.

Next, the VCF file is converted into the VCF2CAPS format (V2C) containing a summary information about each polymorphic sequence locus (Fig 2). The first line of both the index file and the V2C file contains the MD5 hash of the reference and the VCF file, respectively, preceded by the hash (#) sign. When loading a reference and a VCF file, VCF2CAPS checks whether their corresponding processed files (the index file and the V2C file) are already present in the program’s directory. For this purpose the MD5 hash is calculated for the input files (FASTA reference and VCF) and compared to the MD5 hash present in the processed files. If both strings are the same, VCF2CAPS will not proceed to file processing, but it will use the already available processed file(s).

Each record stored in a V2C file is composed of three lines characterized below.

1. The first line contains the greater-than (**>**) sign followed by the locus (polymorphism) ID which is a merge between the sequence name and the polymorphism location within this sequence (both parameters are separated with a colon).
2. The second line contains tab-separated genotypes of all individuals, where 0 corresponds to the allele present in the reference sequence and 1 corresponds to the first alternative allele. In the case of more than one alternative allele– 2, 3, 4, etc. are used, respectively.
3. The third line contains tab-separated fields with information about every identified allele. Each field contains the following comma-separated elements:
  - allele sequence (on Fig 2 the one from the reference is encircled);
  - length of the allele sequence (in bp);
  - allele sequence flanked by two 40 bp reference fragments, e.g. in the case of a SNP the total length of such “extracted” sequence is: 40+1+40 = 81*bp*, in the case of a 10 bp insertion allele the total length of such sequence would be: 40+10+40 = 90*bp*, the extracted allelic sequences are used by VCF2CAPS to search for restriction cut sites (see below).

### CAPS mining

**Fig 3:**
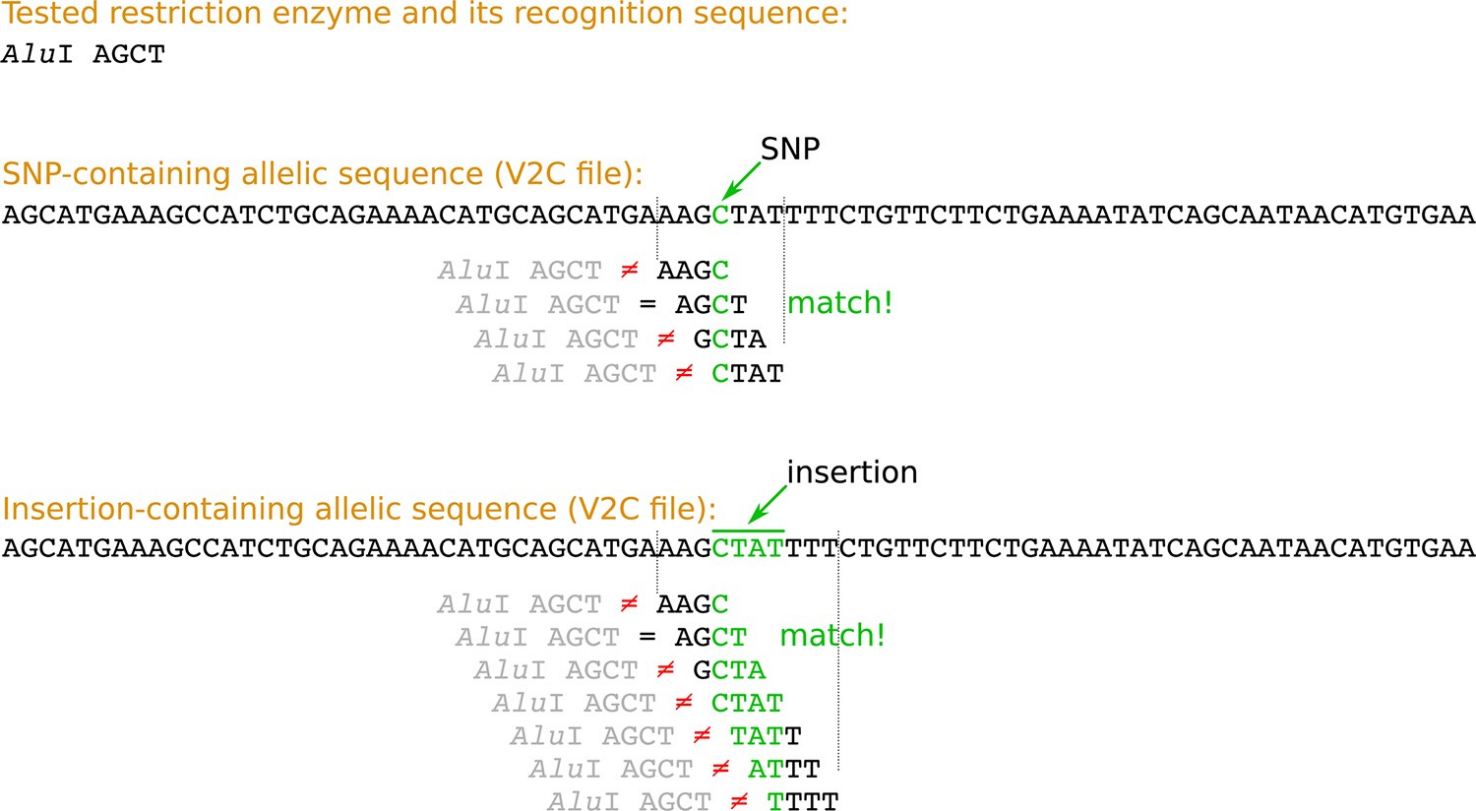
Search for restriction sites by VCF2CAPS.

The CAPS mining function checks every identified allele for its overlap with sequences recognized by restriction enzymes. For this purpose, every allelic sequence taken from the V2C file is cut into fragments of length equal to the length of the currently analyzed restriction site. Subsequently, the obtained set of component allelic fragments is being compared to this particular restriction site (Fig 3).

**Fig 4:**
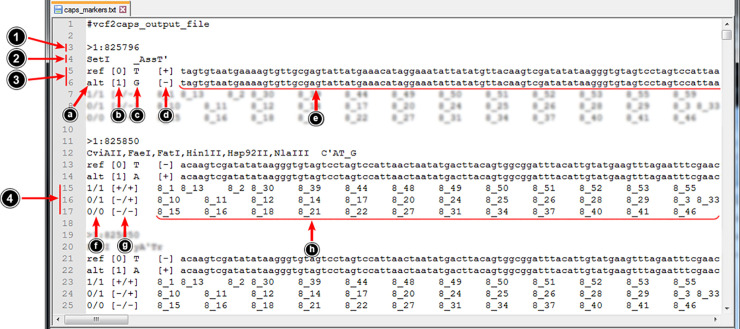
Fragment from an exemplary VCF2CAPS output file–its elements are marked with arrows and described in the text.

An allele, which can be digested by some specific enzyme, is searched among analyzed individuals to find out whether they exhibit polymorphism of the respective locus with both digested and non-digested alleles. If so, the information about the detected alleles and the corresponding restriction enzyme(s) is written to the output file (Fig 4).

Each record stored in the VCF2CAPS output file is composed of four segments characterized below.

1. Single line containing the greater-than (**>**) sign followed by the locus (polymorphism) ID.
2. The second segment is also a single line–it contains the name(s) of a restriction enzyme(s) and its (their) recognition sequence. An apostrophe (’) within such sequence indicates the cut point in the top strand, while an underscore (_) –the cut point in the bottom strand.
3. The third segment contains tab-separated fields with the information about each allele of a given locus (polymorphism):
  - abbreviations ‘ref’ and ‘alt’ correspond to the reference and alternative allele, respectively;
  - allele ID enclosed in square brackets, 0 designates the reference allele and 1, 2, 3, 4 etc. are used for alternative alleles;
  - allelic (variant) sequence, in the case of SNPs and deletion alleles it is only one nucleotide;
  - either a plus or a minus sign enclosed in square brackets indicating whether this specific allele is digested or not by the enzyme(s) specified above;
  - separated by the allelic (variant) sequence (uppercase) fragments of the reference sequence (lowercase) located upstream and downstream from the polymorphic site, the length of these fragments is customizable and can be changed in the software–the default value is 500, e.g. in the case of a SNP the total length of such extracted sequence is: 500+1+500 = 1001*bp*, in the case of a 10 bp insertion allele the total length of the extracted sequence would be: 500+10+500 = 1010*bp*, these extracted sequences can be used for primer design.
4. The fourth segment contains tab-delimited information about genotypes of the analyzed individuals:
  f genotype denoted with the above specified allele IDs e.g. 0/1; undetermined genotypes are denoted as **. /.**;
  g enclosed in square brackets genotype denoted with plus and minus signs corresponding to the digested and non-digested allele, respectively; for undetermined alleles the question mark (?) is put instead; such genotypes correspond to the expected electrophoretic profiles–for amplicons with a single cut site homozygotes −/− should generate a single band corresponding to the non-digested PCR product, for +/+ homozygotes two bands are expected–they correspond to the products of restriction, heterozygotes: −/+ should generate three bands representing all DNA fragments observed for homozygotes;
  h both genotype designations are followed by symbols of the individuals which represent this genotype.

### Filtration of CAPS markers

VCF2CAPS contains two filtration utilities that can be applied to identified CAPS markers.

1 Filtration of CAPS markers with a single cut site for their restriction enzyme(s).

CAPS markers may generate electrophoretic patterns which are more complicated than those outlined above (point 4g). Such complication may result from the presence of additional restriction sites located in the vicinity of the target, polymorphic one. Multi-band, and thus difficult to analyze, gel profiles are produced if these additional cut sites are present within the amplified sequence. For that reason the provided filter keeps only these markers which, within the user-specified sequence length, carry a single restriction site–the target, polymorphic one. The filtered CAPS markers are saved to a new output file with the suffix ’_scf’–its format is the same as for output files without filtration.

2 Filtration of CAPS markers which differentiate user-defined groups of analyzed individuals or samples (filtration by groups).

This filter was designed to simplify identification of CAPS markers which are linked to various traits exhibited by individuals from the analyzed population. The filtering function requires two data inputs: 1) VCF2CAPS output file and 2) two or three groups of analyzed individuals which need to be differentiated by the filtered CAPS markers. The groups are specified in the provided text fields by writing/pasting into them symbols of the belonging individuals. As a result of filtration marker genotypes (represented by the respective individuals) become assigned to the user-specified groups.

**Fig 5:**
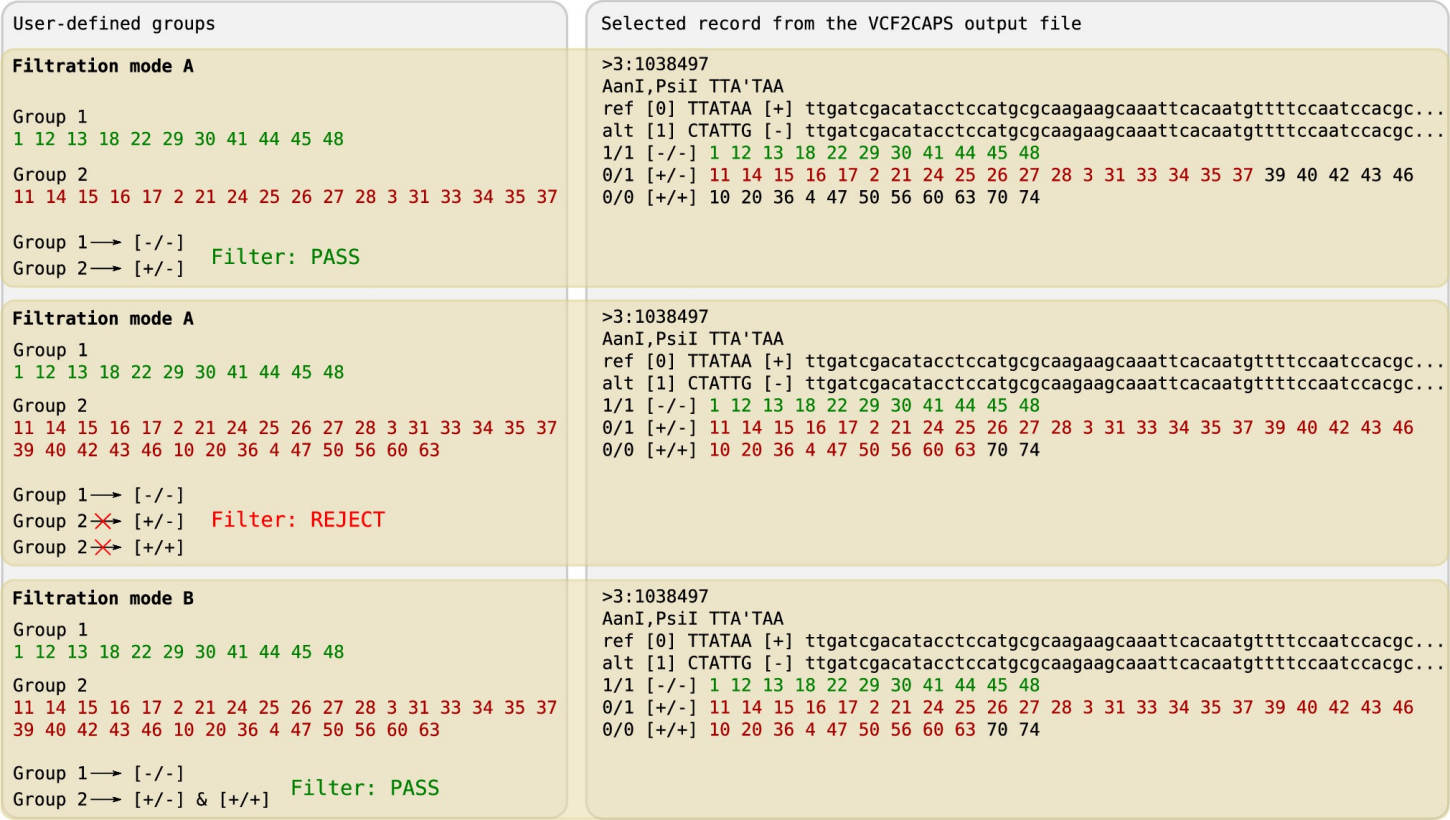
Filtration of CAPS markers with desired distribution of analyzed individuals among detected genotypes (filtration by groups).

The filter can work in two different modes (Fig 5):

- mode A–it will return CAPS markers with one genotype per one user-defined group–through this, each of these groups will be assigned to a specific marker genotype; mode A is designed for cases in which the number of possible user-defined (typically phenotypic) groups equals the number of marker genotypes, e.g. for F_2_ populations with co-dominance or incomplete dominance, and for BC_1_ populations with dominance;
- mode B–it returns CAPS markers with one of the homozygotes (either +/+ or −/−) represented by individuals from one of the two user-defined groups, the remaining individuals (being opposite homozygotes and heterozygotes) should fall into the second user-defined group; therefore, this mode should be used for cases with two user-defined (phenotypic) groups and three marker genotypes–it is designed for F_2_ populations with dominance in which there are two phenotypes/user-defined groups (one represented by dominant homozygotes and heterozygotes, the other by recessive homozygotes) and three marker genotypes (+/+, −/−, +/−).

Individually for each group users can set the matching threshold–i.e. percentage of inconsistent individuals between a given user-defined group and its relevant marker genotypes. In this type of filtration, the returned CAPS markers are saved to a new output file with the suffix ’_gf’–its format is the same as for output files without filtration.

### Export of marker sequences to FASTA format

VCF2CAPS has been designed to identify restriction cleavable alleles within polymorphic loci. In order to utilize these data in CAPS marker technology, the identified polymorphic regions need to be PCR-amplified with appropriate oligonucleotide primers. To facilitate primer design, VCF2CAPS is able to export such sequence regions in FASTA format. This function extracts fragments of the reference sequence with the variant nucleotide(s) in the center. The extracted sequences are of the same length as the sequences present in VCF2CAPS output files (as mentioned before, this length can be adjusted by the user). For a given (polymorphic) locus only one allelic sequence is extracted–the one from the reference. Exported FASTA files can be used as input files for BatchPrimer3 ([30], https://probes.pw.usda.gov/batchprimer3)–a high-throughput web tool for picking PCR primers, it allows processing of up to 500 sequences at a time.

## Results

### Characteristics of sequence data

Genotyping-by-sequencing (GBS) was performed for table beet plants (*Beta vulgaris* L. subs. *vulgaris* Conditiva Group) from population 398 which segregated with respect to fertility restoration (please refer to section *Plant material*). Sequencing of the GBS library produced 293.7 mln reads with 28.4 Gbp of sequence data and an average of 3.7 mln reads per sample (plant). On average, 95.3% of the total bases had the Phred quality score of at least 30. The mean GC content in the obtained reads was 44%. Per plant approximately 96.1% of the reads were aligned to the reference genome (acc. no. AYZS00000000.2)–in total they covered about 3.3% of the published assembly. The mean depth of coverage was 0.7 over the whole genome and 21 over its sequenced portion. The breadth of coverage at 5× depth was 1.5%.

Analysis of the BAM files with Platypus revealed a total of 335,822 high quality, bi-allelic sequence polymorphisms with 265,247 single nucleotide polymorphisms (SNPs), 40,791 multiple nucleotide polymorphisms (MNPs) and 29,784 indels. The average number of missing genotypes per polymorphism was 68.1%.

The most prevalent SNPs were transitions (A↔G or C↔T) (60.4%) while transversions (A↔C, A↔T, C↔G or G↔T) accounted for 39.6%. The least common type of variation was C↔G conversion which represented only 5.7% of all polymorphisms (Table 2).

**Table 2:** Statistics of sequence polymorphisms identified in population 398 of table beet.

Type of polymorphism	Number	Percent
**Total**	335,822	100.0
**Indels**	29,784	8.9
**Multiple nucleotide polymorphisms (MNPs)**	40,791	12.1
**Single nucleotide polymorphisms (SNPs)**	265,247	79.0
**Transitions**		
**A↔G**	80,199	23.9
**C↔T**	79,900	23.8
**Transversions**				
**A↔C**	28,221	8.4
**A↔T**	29,488	8.8
**C↔G**	19,086	5.7
**G↔T**	28,353	8.4

**Fig 6:**
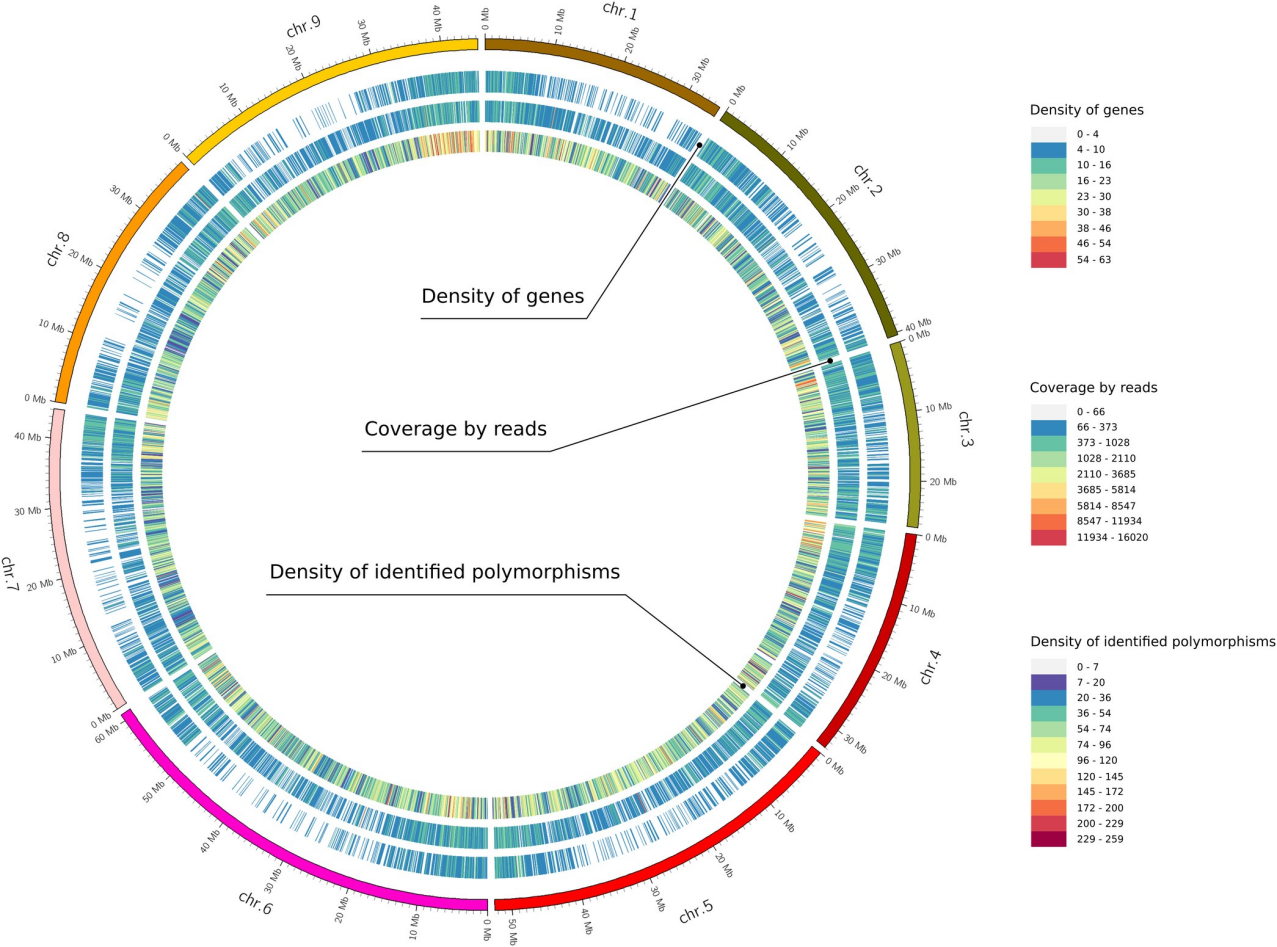
*Beta vulgaris* L. chromosomes overlaid with heatmaps showing gene density, coverage by reads and polymorphism density. The latter two parameters were determined in the presented here GBS experiment. All parameters were calculated using a 100 kb window. The data were visualized using Circos [31].

The number of polymorphisms identified on each chromosome was highly correlated (*r*^2^ = 0.92) with its physical length. Distribution of polymorphisms showed their slightly higher incidence towards the chromosome ends. This distribution correlates with chromosome coverage by reads (Fig 6), which is related to the fact that GBS targets mostly gene-rich regions, such as chromosome ends, by the use of methylation-sensitive restriction enzymes (e.g. *Ape*KI used in this study).

Among the identified polymorphisms, 14.4% were located within the annotated coding sequences (Table 3). Within this pool 61.7% of all transitions were synonymous, while 61,5% of transversions were non-synonymous. Elimination of stop codons was associated with 0.1% of SNPs and 0.1% of indels. Within coding regions most of indels (61.1%) and almost all MNPs (99.4%) did not result in frameshifts.

**Table 3:** Statistics of polymorphism annotation. Percentages were calculated only for polymorphisms located within the annotated coding regions.

Location / Effect	Type of polymorphism			
**SNPs** **MNPs** **Indels**				
**Non-coding regions**	221,790		36,846	28,872
**Coding regions**	43,457		3,945	912
**Stopgain**	1.3%		0.0%	1.6%
**Stoploss**	0.1%		0.0%	0.1%
**Frameshift**	–		0.1%	36.6%
**Non-frameshift**			99.4%	61.1%
**Transitions** **Transversions**			–	
**Non-synonymous**	37.0%	61.5%		
**Synonymous**	61.7%	36.1%		

### Detection of CAPS markers

Out of 335,822 high quality, bi-allelic polymorphisms, 37,643 (11.2%) had not more than 5 missing genotypes. Within this pool, 6,622 polymorphisms displayed segregation expected for an F_2_ population (1:2:1). Using VCF2CAPS all these 6,622 polymorphisms (Table 4) were checked for their overlap with recognition sites of 619 commercially available restriction enzymes. Most of the sequence polymorphisms in the input file were SNPs (80.4%), while MNPs and indels accounted for 7.9% and 11.7% of the entire set, respectively. In total, 5,550 (83.8% of the analyzed pool) polymorphisms were converted into 16,093 CAPS markers (DNA polymorphism–restriction site combinations). Therefore, on average 2.9 polymorphic recognition sites were found for one sequence polymorphism. As could be expected based on the input file, polymorphic recognition sites were delivered mostly from SNPs (82.5%) (Table 4). CAPS-convertible fraction reached 86.1, 95.2 and 60.5% for SNPs, MNPs and indels, respectively. Among the identified CAPS markers the top represented restriction enzyme prototypes were: *MspJ*I, *Sge*I, *Fai*I and *Set*I with 1,590, 1,082, 763 and 582 markers, respectively.

**Table 4:** Statistics of DNA polymorphisms in the VCF2CAPS input and output files generated for population 398.

CAPS markers	DNA polymorphisms (total)	SNPs	MNPs	Indels
**Input file (VCF)**				
–	6,622	5,321 (80.4%)	526 (7.9%)	775 (11.7%)
**Output file with all CAPS markers**				
**16,093**	5,550	4,580 (82.5%)	501 (9.0%)	469 (8.5%)
**Output file–single-cut**				
**3,331**	2,129	1753 (82.3%)	240 (11.3%)	136 (6.4%)

Out of the 16,093 identified CAPS markers, for 3,331 (20.7%) their constituting recognition site was not found within both 500 bp regions flanking the respective sequence polymorphism. Contribution of SNPs, MNPs and indels in the pool of such single-cut CAPS markers was similar to that before filtration (Table 4). Within the single-cut fraction the top represented prototypes were: *Mae*II, *Tsp*GWI, *Hga*I and *Bsr*I with 52, 51, 40 and 36 markers, respectively.

Generally, the run time of VCF2CAPS was proportional to the number of DNA polymorphisms and analyzed recognition sites. In the case of the analyzed population it took ~18 minutes for the CAPS mining function to check 6,622 polymorphisms for 619 recognition sites on a 2.4 GHz Intel Core 2 Duo PC running Windows 7 Professional OS. Filtration of 16,093 CAPS markers for their single-cut character took ~14 minutes on the same computer. Filtration by groups was taking seconds–for the male-sterile and male-fertile plants from population 398 it revealed 37 CAPS markers which represented 16 DNA polymorphisms.

### Linkage mapping of the identified CAPS markers

**Fig 7:**
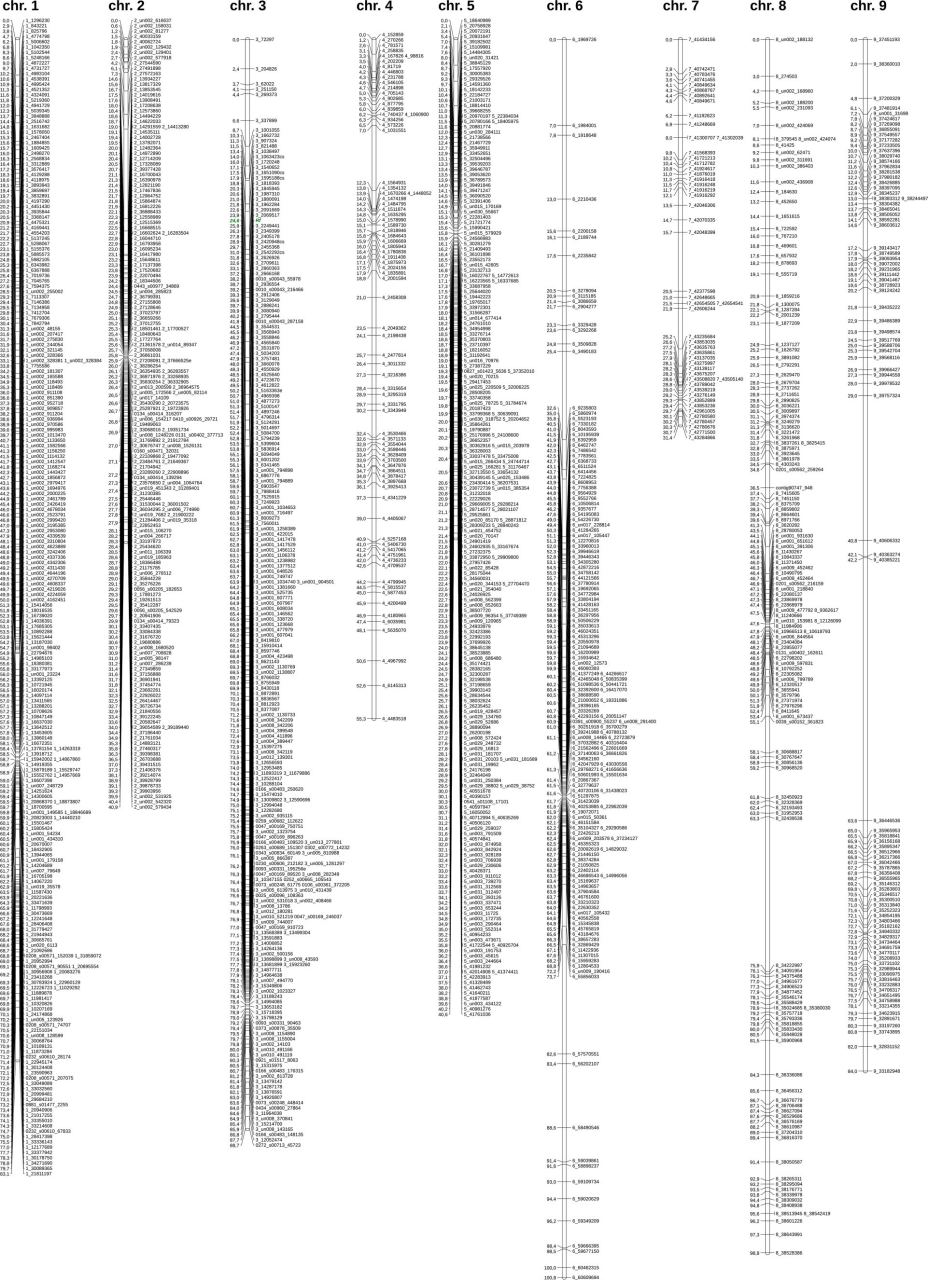
Linkage map based on CAPS-convertible GBS markers from the analyzed segregating population. Most markers are named with a number of the adequate chromosome followed by an underscore and either a nucleotide position of the respective SNP or the first nucleotide of a polymorphic fragment in the case of indels and MNPs. For markers that were not mapped within the genome assembly, the chromosome number is either substituted or supplemented (after underscore) with a relevant scaffold/contig number. Additionally, some markers were marked with *e* or *cs*, where *e* means experimentally verified markers (see next section of the Results), and *cs*–markers co-segregating with the male sterility/fertility phenotype as indicated by the VCF2CAPS filtration utility. *Rf*–restorer gene.

Out of 5,550 CAPS-convertible GBS markers, 1,620 were selected which represented unique sets of genotypes (from groups of ideally co-segregating markers only one was picked up) and were spaced one from another by at least 10 kb (in the reference sequence). From this set of filtered markers, 1,362 and the phenotype (male-sterile vs. male-fertile) were mapped to nine linkage groups/chromosomes (Fig 7). The number of mapped loci ranged from 50 to 231 for chromosome 7 and chromosome 1, respectively (Table 5). The shortest map length– 31.4 cM–was obtained for chromosome 7, and the longest– 100.8 cM–for chromosome 6. The average inter-marker distance varied from 0.2 cM for chromosomes 2 and 5 to 0.9 cM for chromosome 9. The total length of the map was 623.6 cM with an average distance of 0.5 cM between adjacent loci. The restorer gene (*Rf*) was mapped on chromosome 3 along with 213 markers. The *Rf*-flanking markers were mapped 0.7 and 1.3 cM from this locus.

**Table 5:** Parameters of the linkage map based on CAPS-convertible GBS markers from the analyzed segregating population.

Linkage group (chromosome)	Length [cM]	Number of loci	Average inter-marker distance [cM]
**1**	83.1	231	0.4
**2**	40.9	176	0.2
**3**	88.7	214	0.4
**4**	55.3	80	0.7
**5**	40.6	219	0.2
**6**	100.8	157	0.6
**7**	31.4	50	0.6
**8**	98.8	145	0.7
**9**	84.0	91	0.9
**-**	**Total** 623.6	**Total** 1363	**Mean** 0.5

Out of 16 CAPS-convertible GBS markers which co-segregated with the sterility/fertility phenotype (as revealed through filtration by groups–see above) 5 were mapped on chromosome 3 in the vicinity of the *Rf* locus (Fig 7). The remaining 11 markers were filtered out earlier for being too dense (using the 10 kb criterion) or displaying a duplicated segregation pattern.

### Experimental verification of the identified CAPS markers

**Fig 8:**
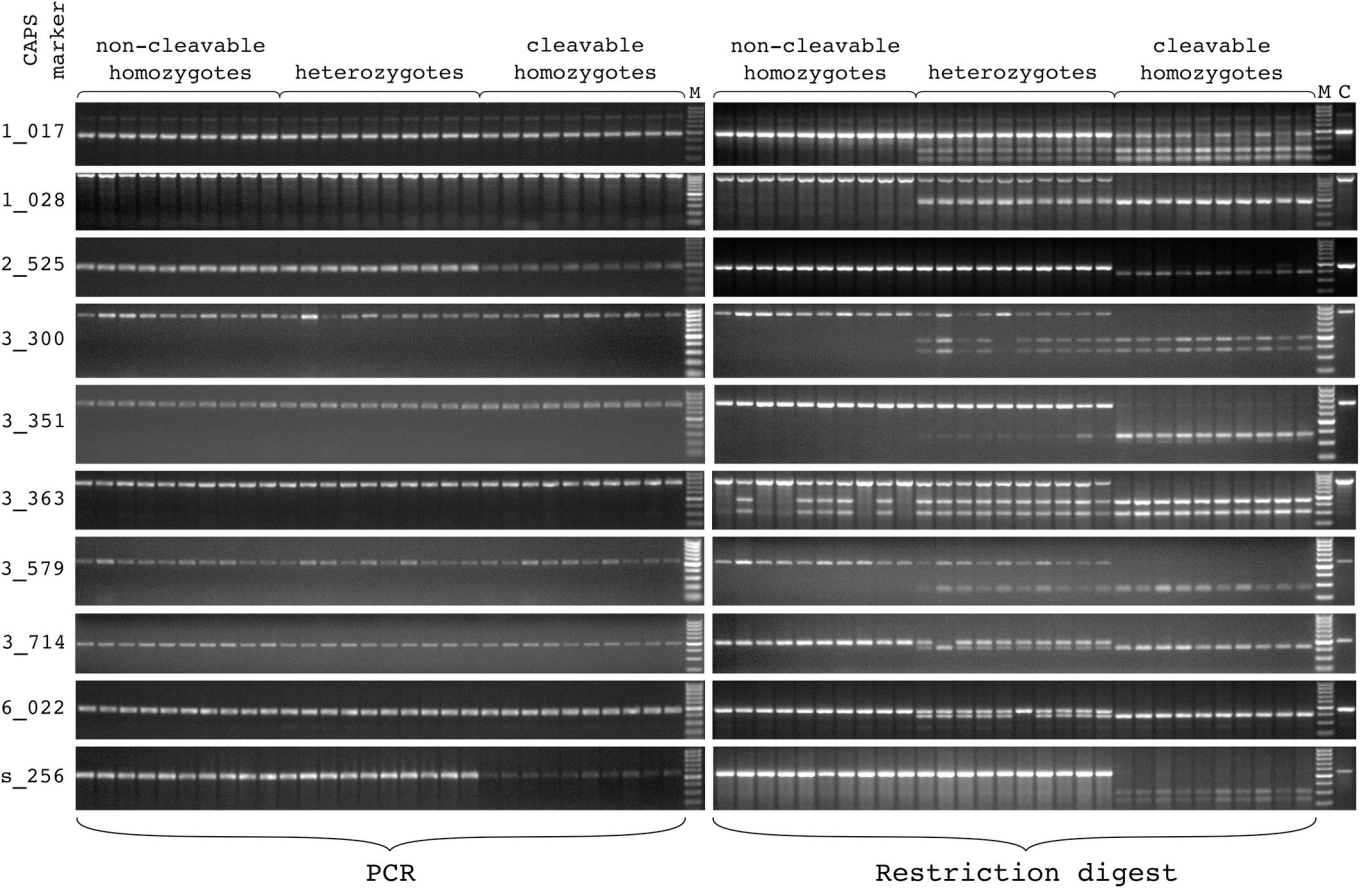
Experimental verification of CAPS markers identified with VCF2CAPS. Analyzed plants originated from population 398 of table beet, and with the program (for a given marker) they were classified as non-cleavable homozygotes, heterozygotes and cleavable homozygotes. Left panel–PCR products, right panel–restriction products (gel regions with some shortest restriction fragments were cropped out of the pictures). In comparison to the previous text and Fig 7 (linkage map) the marker names are abbreviated–only three last digits of the nucleotide position are preserved (Table 1). M–DNA size marker, C–non-digested control.

In order to verify the reliability of VCF2CAPS 10 single-cut markers identified by the program were subjected to experimental validation (Table 1). Each of these markers was tested on 30 plants from population 398 –10 non-cleavable homozygotes, 10 heterozygotes and 10 cleavable (by the restriction enzyme devised for a given marker) homozygotes (Fig 8). For all tested markers the size of the obtained PCR product was accordant with the sequence data, so were the sizes of the derived restriction fragments.

For markers 1_028 and 3_579 distribution of their restriction profiles exactly matched genotypic predictions from the VCF2CAPS output file. Similar situation was observed for marker 3_351, although the band of the cleavable allele was weaker than that of the non-cleavable allele–especially in heterozygotes. Three markers had distribution very close to predictions from VCF2CAPS, the only discrepancy was shown for single supposed heterozygotes which marker-wise turned out to be homozygotes–cleavable in the case of marker 3_714 and non-cleavable for markers 3_300 and 6_022. In the case of marker 3_363 predicted profiles were observed for five supposed non-cleavable homozygotes as well as for all supposed heterozygotes and cleavable homozygotes. This marker showed that the remaining five supposed non-cleavable homozygotes were in fact heterozygotes. Marker 1_017 confirmed the character of the supposed non-cleavable homozygotes and heterozygotes. In turn, the profiles produced for all supposed cleavable homozygotes were similar to those of heterozygotes, although with the cleavage products stronger and the non-cleaved DNA fragment weaker than in heterozygotes. For the remaining two markers– 2_525 and s_256 –the VCF2CAPS predictions were confirmed in all supposed homozygotes (both non-cleavable and cleavable), but all supposed heterozygotes looked like the non-cleavable homozygotes. For these two markers the cleavable homozygotes yielded much weaker amplification products than the remaining plants. This further translated into very weak bands observed after restriction.

## Discussion

In recent years rapid development of new generation sequencing (NGS) has led to invention of genotyping methods which allow for massive identification of DNA polymorphisms, even in very large populations. Such NGS-based genotyping produces huge amounts of sequence data requiring specialized bioinformatic tools to handle. Currently, there is only one, freely-available software–PCR Markers Tools [17]–allowing for SNP-based CAPS marker design from NGS data. It is a set of programs for detection of restriction polymorphisms in GFF3-formatted data and for subsequent PCR primer design. However, this software may not be suitable for all users and for all types of data. This is partially due to the fact that the Galaxy version of PCR Markers Tools requires setting up the Galaxy server on a local computer or in the cloud. In turn, setting up the server needs some experience in informatics and is only available for UNIX/Linux and Mac OSX users. Moreover, for Windows OS users only Python scripts are available and therefore, experience in using console applications is required. Another difficulty stems from the fact that PCR Markers Tools are compatible with VCF files generated by the mpileup utility from SAMtools. Such files have specific data in the INFO field and this information is required by scripts vcf_gff.py and find_CAPS.py to identify CAPS-convertible SNPs (MNPs and indels are not accepted by PCR Markers Tools for conversion into CAPS markers). VCF files created with different SNP-calling software–e.g. TASSEL [32], Platypus [23], FreeBayes [33]–may not include tags in that field. An attempt to identify restriction polymorphisms in such files will fail (with an error message popping up) if they contain variants other than SNPs. This problem may even affect VCF files created using SAMtools mpileup–if they are subjected to filtration using VCFtools. By default, VCFtools removes all INFO tags from its output file unless option–recode-INFO-all is applied. In PCR Markers Tools script find_CAPS.py has twenty hard-coded restriction endonucleases. This set may be sufficient for many projects. However, for applications in which the number of CAPS-convertible DNA polymorphisms is critical, this narrow set of enzymes may pose a problem. The Authors of PCR Markers Tools declared that the enzyme list can be modified/extended–however, it requires editing of the source code and likely this task is too demanding for less experienced users.

To overcome the described limitations, we have developed a new computer program named VCF2CAPS, which serves for identification of polymorphic restriction recognition sites among variants stored in VCF files. It has a simple and intuitive graphical user interface (GUI). VCF2CAPS was written in the Perl 5 programming language, so it can be run on UNIX/Linux, Windows and Mac OSX (not tested) operating systems with the Perl environment installed on them. For Windows users, who are not accustomed to working with console applications (this may be needed to install the required Perl modules) we provide an executable file–VCF2CAPS.exe–that can be directly launched after downloading from the GitHub repository. Another advantage of our program is that it is not dependent on tag values within the input file–therefore, it should accept any VCF file created with any variant-calling software. Additionally, in contrast to PCR Markers Tools, VCF2CAPS is able to identify restriction polymorphisms resulting not only from SNPs but also from MNPs and indels. This feature can substantially enlarge the output–in our data the fraction of MNP- and indel-based CAPS markers reached almost 17.5%. Another useful improvement in VCF2CAPS is that it can search for polymorphic recognition sites of all restriction enzymes included in REBASE [29].

VCF2CAPS has been also equipped with a unique filtration utility–it allows quick identification of markers for which segregation of genotypes matches the user-defined groups of individuals (samples). If such groups correspond to phenotypic classes this filtration will return markers linked to the trait of interest. Within our dataset, VCF2CAPS identified 16 CAPS-convertible GBS markers which differentiated male-sterile from male-fertile (restored) plants. Such pre-selected markers can be further tested on other segregating populations to verify linkage with the gene which is responsible for the analyzed trait. In this way filtration by groups within VCF2CAPS can partially substitute the traditional procedures of genetic mapping, although in principle it is not restricted to the linkage context–e.g. it can be used for identification of markers which distinguish populations of different origin.

Evaluation of VCF2CAPS was performed with the use of a genotyping-by-sequencing (GBS) dataset produced for an F_2_ table beet population. Despite the presence of the sterilizing (S-) cytoplasm this population segregated into male-fertile and male-sterile plants–their ratio (3:1) showed that fertility was conditioned by a single dominant restorer gene. As indicated by parameters like number of reads per sample (individual plant), read depth and quality scores our sequencing data met the standards set by other studies exploiting the GBS technology [34–36].

Analysis of the mapped reads with Platypus [23] lead to identification of 335,822 bi-allelic polymorphisms–the majority of them were located in non-coding sequences (85.6%), but mostly within gene-rich parts of the genome. Such distribution agreed with expectations based on the fact that the enzyme used for library preparation–*Ape*KI–was sensitive to CpG methylation [37]. The high number of identified biallelic polymorphisms ensured that even after their very stringent filtration (down to 0.48% of the initial pool) it was possible to construct a high density linkage map based on CAPS markers. This is the first high-density genetic map of table beet–previously such maps have been developed for sugar beet and for sugar beet × table beet crosses [38–44]. On our map the average distance between markers is 0.5 cM making it one of the densest genetic maps of beet (even though only a subset of CAPS-convertible polymorphisms was used for mapping). Within our linkage groups the mapped loci tend to form clusters either in the center or in the distal parts–best examples are groups 6 and 9, respectively. Such differences in marker density (presence of clusters and gaps) are quite common in maps based on massively produced SNPs [45]. Likely, this may result from the fact that high-throughput genotyping methods (including GBS) target mostly gene-rich regions of the genome [46]. Our map incorporated 309 markers from scaffolds and contigs which were not yet included in the *Beta vulgaris* L. genome assembly. Therefore, it is possible that the obtained linkage information will be useful in further improvements of this assembly.

In order to test if markers identified with VCF2CAPS were useful for trait mapping, the population chosen for analysis segregated into male-sterile and male-fertile (restored) plants. This enabled fine mapping of the fertility restorer gene from table beet. The restorer clearly mapped to chromosome 3 indicating that it corresponded to the sugar beet *X*/*Rf1* gene identified already by Owen [47,48] and recently cloned by Matsuhira et al. [49]. This indication was further supported by co-localization of the genetically closest CAPS markers and four paralogous ORFs of *Rf1* within the sequence of chromosome 3. The shortest physical distance between the quadruplicated ORF and one of our CAPS markers was approx. 4 kb. Such proximity is very promising for practical exploitation (marker-assisted selection)–the more so because markers from both sides of the restorer gene were identified.

To test overall accuracy of marker predictions produced by VCF2CAPS a set of 10 markers was chosen for experimental validation. In general, the gel data matched the genotypes from the program output file and all observed inconsistencies could be attributed to shortcomings of either GBS or CAPS analysis. GBS can be blamed for misgenotyping of particular plants which were correctly classified using CAPS analysis. This applies to single homozygote plants which were found with markers 3_300, 3_714 and 6_022 within the pool of GBS-inferred heterozygotes. Likely, such an effect arises when both correct and erroneous reads are mapped to the reference, particularly in the context of low sequencing depth which is typical for GBS [37]. Probably low sequencing depth was also responsible for the inconsistency revealed with marker 3_363 – 50% of supposed (based on GBS) non-cleavable homozygotes were in fact heterozygotes. Apparently, only one allele was represented in sequencing reads produced for those misgenotyped heterozygotes–one can expect that with better genome coverage both sequence variants would be detected. This, however, does not explain why the bias was observed only towards one (non-cleavable) allele. Some reports point at library preparation, especially the amplification step, as a potential source of coverage variation (including underrepresentation of certain sequences) in NGS ([50] and references therein). Most of the remaining inconsistencies can be explained by the effect of allele dropout during PCR in the course of CAPS analysis. This phenomenon leads to misidentification of heterozygotes as homozygotes and it is considered very challenging even in carefully optimized clinical and forensic diagnostics [51,52]. Preferential amplification of the non-cleavable allele was already observed for marker 3_351 which otherwise produced profiles perfectly matching the GBS data. However, in the case of two other markers– 2_525 and s_256 –the PCR bias was strong enough to make heterozygotes looking like non-cleavable homozygotes, although the true cause of this discrepancy can be deduced from very poor amplification observed in cleavable homozygotes. Among other possible reasons, allelic preference in PCR may result from mismatches between a primer and its target sequence [53,54] with such situations being quite likely in our assays as sources of the reference genome (it was used for primer design) and template DNA were not the same (sugar beet vs. table beet). Yet another inconsistency was observed with the use of marker 1_017 –cleavable homozygotes yielded profiles similar to those of heterozygotes, although with the non-cleavable DNA fragment looking distinctly weaker. Theoretically, such an effect could have resulted from partial (incomplete) restriction–however, this possibility was excluded after careful optimization of this particular digest. An alternative explanation assumes that the 1_017 amplicons were generated from duplicated loci–one of them represents the assayed polymorphic site (earlier identified with GBS), the other is a weaker amplified off-target sequence which lacks the restriction site. In the light of the above interpretations, all ten sampled markers were correctly predicted with VCF2CAPS, although the value of a given marker was dependent both on the quality of sequence data and correctness of amplicon design. Likely, the PCR-related problems could be overcome by simple redesign of the primers, and moreover, the universal remedy for all suboptimal assays is redundancy of markers identified from NGS data.

## Availability and future directions

VCF2CAPS is an open-source software, freely available under the terms of the GNU General Public License (GPL) v.3.0 (https://www.gnu.org/licenses/gpl-3.0.html). The Perl script of VCF2CAPS as well as its MS Windows executable version can be downloaded from GitHub (https://github.com/Aviatore/vcf2caps). The program is provided together with VCF exemplary files and a manual. Running the program directly from the script requires Perl v.5.008 or later.

We plan to improve performance of the algorithm by implementing concurrency/multithreading. Further optimization of VCF2CAPS could be achieved by rewriting its code from Perl to C#. Likely, this would also have a positive impact on code maintainability.
